# PARP Inhibition Increases the Reliance on ATR/CHK1 Checkpoint Signaling Leading to Synthetic Lethality—An Alternative Treatment Strategy for Epithelial Ovarian Cancer Cells Independent from HR Effectiveness

**DOI:** 10.3390/ijms21249715

**Published:** 2020-12-19

**Authors:** Patrycja Gralewska, Arkadiusz Gajek, Agnieszka Marczak, Michał Mikuła, Jerzy Ostrowski, Agnieszka Śliwińska, Aneta Rogalska

**Affiliations:** 1 Department of Medical Biophysics, Faculty of Biology and Environmental Protection, Institute of Biophysics, University of Lodz, 90-236 Lodz, Poland; patrycja.gralewska@edu.uni.lodz.pl (P.G.); arkadiusz.gajek@biol.uni.lodz.pl (A.G.); agnieszka.marczak@biol.uni.lodz.pl (A.M.); 2 Department of Genetics, Maria Sklodowska-Curie National Research Institute of Oncology, 02-781 Warsaw, Poland; michal.mikula@pib-nio.pl (M.M.); jerzy.ostrowski@pib-nio.pl (J.O.); 3 Department of Gastroenterology, Hepatology and Clinical Oncology, Centre of Postgraduate Medical Education, 01-813 Warsaw, Poland; 4 Department of Nucleic Acid Biochemistry, Medical University of Lodz, 92-213 Lodz, Poland; agnieszka.sliwinska@umed.lodz.pl

**Keywords:** ATR inhibitor, CHK1 inhibitor, ovarian cancer, PARP inhibitor, targeted therapy

## Abstract

Poly (ADP-ribose) polymerase inhibitor (PARPi, olaparib) impairs the repair of DNA single-strand breaks (SSBs), resulting in double-strand breaks (DSBs) that cannot be repaired efficiently in homologous recombination repair (HRR)-deficient cancers such as BRCA1/2-mutant cancers, leading to synthetic lethality. Despite the efficacy of olaparib in the treatment of BRCA1/2 deficient tumors, PARPi resistance is common. We hypothesized that the combination of olaparib with anticancer agents that disrupt HRR by targeting ataxia telangiectasia and Rad3-related protein (ATR) or checkpoint kinase 1 (CHK1) may be an effective strategy to reverse ovarian cancer resistance to olaparib. Here, we evaluated the effect of olaparib, the ATR inhibitor AZD6738, and the CHK1 inhibitor MK8776 alone and in combination on cell survival, colony formation, replication stress response (RSR) protein expression, DNA damage, and apoptotic changes in BRCA2 mutated (PEO-1) and HRR-proficient BRCA wild-type (SKOV-3 and OV-90) cells. Combined treatment caused the accumulation of DNA DSBs. PARP expression was associated with sensitivity to olaparib or inhibitors of RSR. Synergistic effects were weaker when olaparib was combined with CHK1i and occurred regardless of the BRCA2 status of tumor cells. Because PARPi increases the reliance on ATR/CHK1 for genome stability, the combination of PARPi with ATR inhibition suppressed ovarian cancer cell growth independently of the efficacy of HRR. The present results were obtained at sub-lethal doses, suggesting the potential of these inhibitors as monotherapy as well as in combination with olaparib.

## 1. Introduction

Ovarian cancer is the most common cause of death among gynecological cancers, which is partly attributed to the fact that epithelial ovarian cancer (EOC) is often diagnosed at an advanced stage. The risk of ovarian cancer is associated with DNA damage [1]. Most type II ovarian carcinomas (96%) have TP53 mutation [2,3] and approximately 50% of germline high-grade serous ovarian cancers (HGSOCs) carry an alteration in homologous recombination (HR) pathway genes, most commonly in breast cancer (BRCA) gene 1/2 [4]. Moreover, DNA damage repair genes are mutated in 40.3% of patients with HGSOC, and the rate of somatic BRCA mutations in the absence of germline mutation is 8.5% [5]. Therefore, targeting DNA repair-associated proteins is increasingly becoming an applicable approach to cancer treatment. Single-strand breaks (SSBs) can be accurately repaired using the other strand as a template, and this process involves the poly (ADP-ribose) polymerase (PARP) enzyme [6]. DNA double-strand breaks (DSBs) are repaired through two pathways: the HR pathway and non-homologous end joining, although other mechanisms also exist [7]. BRCA1 and BRCA2 are involved in the DNA damage response; they are important members of the network of interacting DNA repair pathways responsible for the maintenance of genome stability. Both proteins are related to the error-free repair of DSBs by HR [8]. BRCA1 signals DNA damage and ensures cell cycle regulation, whereas BRCA2 interacts with RAD51 and facilitates the loading and formation of RAD51 filaments on the damaged DNA strand [9]. HR-deficient cancer cells with mutations in BRCA1 or BRCA2 are associated with DSB repair via error-prone repair pathways, the accumulation of mutations, and cell death [10]. PARP inhibitors (PARPi) impair SSB repair, thereby causing DSBs that cannot be repaired efficiently in BRCA1/2-mutant cancers, leading to synthetic lethality [11]. The first FDA-approved PARPi was olaparib (Lynparza). In 2014, olaparib gained European Medicines Agency approval for the treatment of advanced EOC in patients with germline BRCA^MUT^ who did not respond to at least three lines of chemotherapy [12]. Olaparib is currently the only FDA-approved PARPi for first-line maintenance therapy in BRCA^MUT^ patients [13]. Despite the efficacy of olaparib in the treatment of BRCA1/2 deficient tumors, the development of resistance to PARPi is common. It is known that PARP1 is altered in 1.12% of high-grade ovarian serous adenocarcinoma patients and in 1.05% of ovarian carcinoma patients [14]. Mutations of the PARP1 DNA-binding zinc-finger (ZnF) domains cause PARPi resistance and can alter PARP1 trapping [15]. Although olaparib treatment increases the survival rate of patients with stage IV disease, the 5-year survival rate of patients with advanced stage cancer remains below 30% [16].

ATR/CHK1 blockade prevents DNA damage-induced cell cycle arrest, resulting in inappropriate entry into mitosis, chromosome aberrations, unequal partitioning of the genome, and cell apoptosis [17]. The ATR/CHK1 pathway stabilizes replication forks and prevents their collapse into DNA DBSs. Phosphorylation of ATR at Thr1989 occurs in response to DNA damage and is important for the activation of the ATR/CHK1 pathway. Replication protein A (RPA), ATR-interacting protein (ATRIP), and ATR kinase are involved in Thr1989 phosphorylation [18]. Replication blocks and genotoxic stress induce CHK1 phosphorylation at Ser317 and Ser345. Combination treatment with olaparib and anticancer agents that disrupt HR repair such as ATR inhibitors (ATRi) or CHK1 inhibitors (CHK1i) may therefore represent an effective strategy to sensitize ovarian cancer cells to olaparib. Thus, the inhibition of ATR/CHK1 is expected to increase reliance on HR to reorganize the replication fork structure and complete the replication. ATR is a key regulator of DNA replication stress response (RSR) and DNA damage-activated checkpoints. Together with CHK1, ATR plays a role in M-phase inducer phosphatase (CDC25) phosphorylation and inactivation [19]. The ATR kinase lies upstream of its effector protein, CHK1 [20], and phosphorylates numerous factors, including Werner syndrome ATP-dependent helicase (WRN), SWI/SNF-related matrix-associated actin-dependent regulator of chromatin subfamily A-like protein 1 (SMARCAL1), and Fanconi anemia complementation group I (FANCI), which may help preserve replication fork stability and control cell cycle progression [21,22,23]. Additionally, replication protein A (RPA), DNA replication licensing factor (MCM2), and p53 are the direct substrates of ATR [24]. Thus, ATR is essential for cell survival, as it has a crucial role in stabilizing genomic integrity [25].

ATR inhibition is lethal in numerous ovarian cancer cell lines associated with changes in TP53 (OV-90) or BRCA1/2 (PEO-1, BRCA2^MUT^) [26,27]. Oncogene expression (oncogenic RAS protein mutations and MYC proto-oncogene protein and G1/S-specific cyclin-E1 overexpression) and other defects such as deficiencies in DNA repair (PALB2 and the ataxia telangiectasia mutated (ATM) loss) can cause DNA replication stress and increased reliance on the ATR [28]. The activation of CHK1 and its downstream effectors leads to coordinated activities, including reduced new origin firing, delay of cell cycle progression, and restoration of the stalled replication forks [29]. CHK1 is an important molecular target for sensitizing cancer cells to DNA-damaging agents. In response to DNA damage, CHK1 inhibits the Cdc25 phosphatase, resulting in cyclin-dependent kinase (Cdk) inhibition and cell cycle arrest in the G2 phase mainly in p53 mutant tumor cells. Therefore, CHK1 inhibition renders cells more sensitive to DNA damage and programmed cell death [17].

Herein, we showed that PARPi treatment increased ATR and CHK1 phosphorylation, a molecular step crucial for the activation of the ATR/CHK1 fork protection pathway. We also investigated the efficacy of the combination of PARPi with ATRi or CHK1i in BRCA^MUT^ ovarian cancer cells, as well as in serous ovarian cancers without BRCA1/2 mutation.

## 2. Results

### 2.1. PARP Inhibition Is Not Sufficient to Kill Ovarian Cancer Cells, but Acts Synergistically with CHK1 or ATR Inhibition

**Figure 1:**
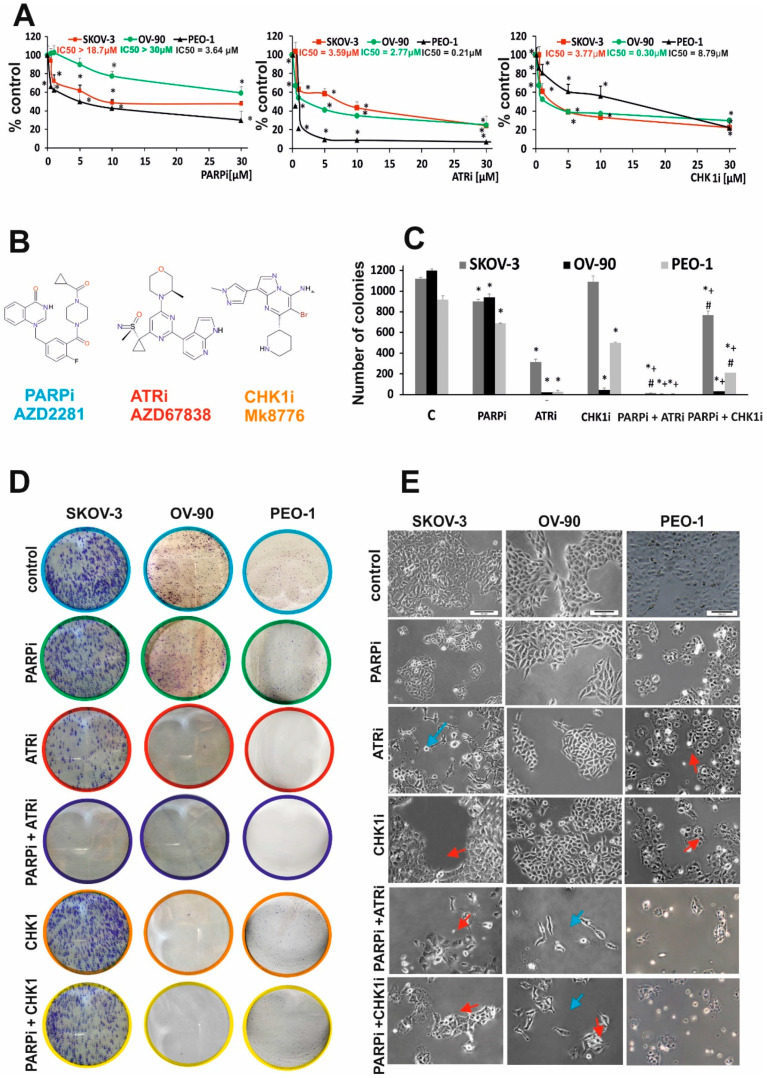
PARPi in combination with CHK1i or ATRi decreased viability more effectively than PARPi monotherapy. (**A**) Cell viability after treatment with PARPi (AZD2281), CHK1i (MK8776), and ATRi (AZD6738) in HR-deficient (PEO-1, BRCA2^MUT^) and HR-proficient (OV-90, SKOV-3, BRCA^WT^) cells at increasing concentrations for 5 days was assessed by the MTT assay. (**B**) Structural formulas of PARPi, ATRi, CHKi. (**C**,**D**) Colony formation assay. Colony formation was evaluated after treatment with PARPi at 0.5 µM in SKOV-3, OV-90, and PEO-1 cells. Cells (200 cells/well) were seeded into six-well plates and incubated with the indicated drug concentrations for 14 days. Colony numbers were counted manually (* *p* < 0.05). For each sample, the results from three replicates were averaged. PARPi and CHK1i decreased viability to 80.17% and 97.28%, respectively, compared with the control SKOV-3 cells; to 78.33% and 3.63%, respectively, in OV-90; and to 75.35% and 0.25%, respectively, in PEO-1 cells. Combination therapy with PARPi and CHK1i decreased colony formation to 68% in SKOV-3, 2.5% in OV-90, and 22.82% in PEO-1 cells. In all cell lines, drugs used in combination had a synergistic effect compared with single drug administration (SKOV-3, CDI = 0.07; OV-90, CDI = 0.07; PEO-1, CDI = 0.06). Combined PARPi and ATRi treatment decreased colony formation to <1% compared with PARPi alone and ATRi alone. In all cell lines, the combination of PARPi/ATRi had a synergistic effect compared with single compounds (SKOV-3, CDI = 0.004; OV-90, CDI = 0.03; PEO-1, CDI = 0.01). All data correspond to three biological assays and were graphed as means ± SD. (**E**) The morphology of SKOV-3, OV-90, and PEO-1 cells treated for 24 h with ATRi, CHK1i, and their combination with olaparib (0.5 µM concentration of each single drug) was examined under an inverted microscope (Olympus IX70, Japan) (scale bar = 100 μm). The cells were elongated and thin (blue arrows) or enlarged (red arrows). * Statistically significant changes between samples incubated with the compound compared with control cells (*p* < 0.05). + Statistically significant changes between samples incubated with PARPi and combination treatments (PARPi/ATRi; PARPi/CHK1i) (*p* < 0.05). # Statistically significant differences between samples incubated with ATRi or CHKi and their combination (PARPi/ATRi; PARPi/CHK1i) (*p* < 0.05).

The antiproliferative activity of the compounds was assessed using the 3-(4,5-dimethylthiazol-2-yl)-2,5-diphenyltetrazolium bromide (MTT) assay. Olaparib (AZD2281) had a dose-dependent effect and was more cytotoxic in BRCA^MUT^ cells (PEO-1) than in HR-proficient cells (OV-90 and SKOV-3 cells, BRCA^WT^), as indicated by the IC50 values (Figure 1A). PARPi did not cause complete cell death in SKOV-3 cells (48% of viable cells) or in OV-90 cells (60% of viable cells), which was also observed in BRCA^MUT^ cells (29% of the cells still viable) after 5 days of incubation. We therefore examined the effect of PARPi on the ATR/CHK-1 pathway. The CHK1i MK8776 was added in increasing concentrations and was more cytotoxic in HR-proficient cells (OV-90, SKOV-3) than in BRCA^MUT^ cells (PEO-1) after 5 days of treatment. The ATRi AZD6738 caused a dose-dependent decrease in cell viability in all cell lines, although its effect was stronger in BRCA^MUT^ (PEO-1) than in BRCA^WT^ (OV-90, SKOV-3) cell lines. The IC50 values for ATRi in SKOV-3, OV-90, and PEO-1 were 3.59, 2.77, and 0.21 µM, respectively (Figure 1A).

**Figure 2:**
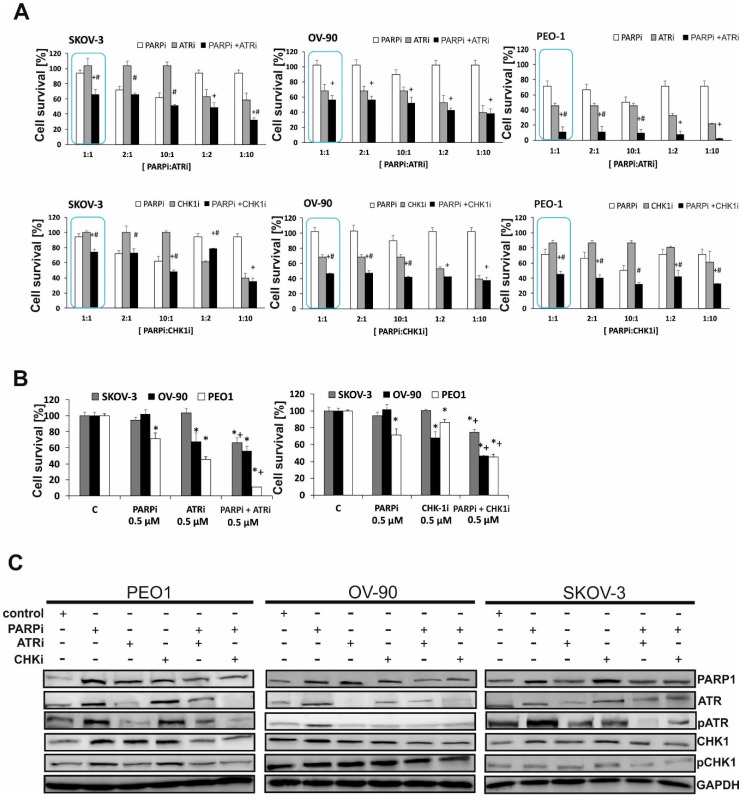
PARPi in combination with CHK1i or ATRi has a synergistic effect in ovarian cancer cells. (**A**) The effect of combination treatment with ATRi or CHK1i and PARPi at different ratios was evaluated by the MTT assay. In all cell lines, the combination of ATRi/PARPi had a synergistic effect compared with either drug alone (SKOV-3, CDI = 0.69; OV-90, CDI = 0.82; and PEO-1, CDI = 0.34). Similar effects were obtained with the combination of CHK1i/PARPi compared with either drug alone (SKOV-3, CDI = 0.79; OV-90, CDI = 0.66; and PEO-1, CDI = 0.74). (**B**) The combination effect of PARPi/ATRi and PARPi/CHK1i at 0.5 µM was evaluated by the MTT assay. * Statistically significant differences between samples incubated with the compound compared with control cells (*p* < 0.05). + Statistically significant changes between samples incubated with PARPi and combination treatment (PARPi/ATRi; PARPi/CHK1i) (*p* < 0.05). # Statistically significant differences between the samples incubated with ATRi or CHKi and combination treatment (PARPi/ATRi; PARPi/CHK1i) (*p* < 0.05). (**C**) BRCA2^MUT^ (PEO-1) and HR-proficient (SKOV-3 and OV-90) cells were treated with PARPi, ATRi, CHK1i, and the combination of PARPi/ATRi or PARPi/CHK1i at 0.5 µM, and lysates were collected at 24 h. Western blot analysis of phosphorylated and total proteins was performed. PARP increased with PARPi by 4.9-fold in PEO-1 cells, 2.52-fold in OV-90 cells, and 2.3-fold in SKOV-3 cells. PARPi with ATRi decreased PARP by 1.43-fold in PEO-1 cells, 1.3-fold in OV-90 cells, and 1.1-fold in SKOV-3 cells. PARPi with CHK1i decreased PARP by 1.7-fold in PEO-1 cells, 1.6-fold in OV-90 cells, and 1.1-fold in SKOV-3 cells. ATRi treatment decreased pATR 1.4-fold in PEO-1 cells, 1.34-fold in OV-90 cells, and 2.36-fold in SKOV-3 cells. PARPi increased pATR, whereas the combination of PARPi/ATRi decreased pATR by 2-fold in PEO-1 and OV-90 cells and 1.8-fold in SKOV-3 cells. With CHK1i, pCHK1 increased by 1.36-fold in PEO-1 cells, 1.34-fold in OV-90 cells, and 1.17-fold in SKOV-3 cells. PARPi with ATRi decreased pCHK1 by 1.8-fold in PEO-1 cells and 1.18-fold in SKOV-3 cells compared with PARPi alone. PARPi with CHK1i decreased pCHK1 by 2.95-fold in PEO-1 cells, 1.5-fold in OV-90 cells, and 1.25-fold in SKOV-3 cells compared with PARPi alone.

The in vitro effect of single drug or combined treatment with PARPi and ATRi or CHK1i on ovarian cancer cell lines was evaluated by colony formation assay, a reliable test for measuring cell survival based on the ability of a cell to grow into a colony. Treatment with 0.5 µM PARPi decreased the colony-forming ability to a greater extent in BRCA^MUT^ cells than in HR-proficient cells (Figure 1B,C). ATRi completely suppressed colony formation in HGSOCs (PEO-1 and OV-90) and significantly decreased colony numbers in SKOV-3 cells (up to 28%) (Figure 2B,C). An extended incubation period of 14 days with the tested compounds showed that cell lines with mutated BRCA2 (PEO-1) and those with mutated p53 (OV-90) were CHK1i-sensitive. Combined therapy with PARPi and CHK1i decreased colony formation to 74.55% in SKOV-3 (coefficient of drug interaction (CDI) = 0.79), to 46.06% in OV-90 (CDI = 0.67), and to 45.41% in PEO-1 cells (CDI = 0.74). Combined PARPi and ATRi treatment decreased cell viability to 66.22% in SKOV-3, 56.29% in OV-90, and 11.06% in PEO-1 cells compared with the effect of PARPi alone and ATRi alone (*p* < 0.05) (Figure 2B).

Among ovarian cancer cells treated with PARPi, ATRi, or CHK1i for 24 h (Figure 1D), a small percentage of cells exhibited morphological changes such as elongation and thinning. Exposure of HR-deficient and HR-proficient cells to the combined action of PARPi/ATRi or PARPi/CHK1i significantly increased the number of deteriorated, elongated cells. PARPi/ATRi- and PARPi/CHK1i-treated cells showed an increase in volume, which is characteristic of a defective cell division arrest [30]. In addition, exposure to combinations of compounds led to cell disintegration and detachment of cells from the culture well surface.

To determine the most effective ratio of PARPi to ATRi or CHK1i, the effect of multiple-dose combinations was examined as displayed in Figure 2A,B. The 1:1 (0.5 µM: 0.5 µM) combination was the most effective among those tested. Subtoxic concentrations of PARPi had a smaller effect on cell viability in SKOV-3 and OV-90 cells than in PEO-1 cells (71.15 % of control). CHK1i caused a significant loss of viability (68%) in OV-90 cells and resulted in 86% of viable PEO-1 cells.

The effect of PARP inhibition on activating the ATR/CHK1 pathway was demonstrated by Western blotting as shown in Figure 2C. PARPi treatment at 0.5 µM upregulated phospho-ATR (pATR) and phospho-CHK1 (pCHK1) in both BRCA^MUT^ (PEO-1) and HR-proficient cells (OV-90) within 24 h, although it was more effective in BRCA^MUT^ cells, suggesting the activation of ATR/CHK1 for survival. PARPi did not significantly affect the level of pATR and pCHK1 in the SKOV-3 line (HR-proficient cells), whereas it caused a 2.36-fold increase of pATR in PEO-1 and 1.3-fold increase in OV-90. In PEO-1 and OV-90 cells, pCHK1 increased 1.5- and 1.21-fold, respectively, compared with the controls after 24 h (Figure 2C). In all cell lines, PARPi in combination with ATRi or CHK1i decreased the PARPi-induced upregulation of pATR and pCHK1. CHK1i treatment upregulated pCHK1 in PEO-1 and OV-90 cells.

Despite the dose-dependent cytotoxic effects of olaparib on all ovarian cancer cell lines tested, its activity was not sufficient to significantly reduce cancer cell populations at the concentration range used. Combination treatment with two compounds, PARPi and CHK1i, had a significantly greater cytotoxic effect, decreasing colony formation ability to a greater extent than either drug alone in BRCA2^MUT^ cells compared with BRCA^WT^. ATRi in combination with PARPi was equally cytotoxic in both BRCA2-deficient and wild-type cells. The PARPi/ATRi combination demonstrated synergy in all the tested cell lines, whereas the PARPi/CHK1i combination showed synergism only in BRCA^MUT^ cells.

### 2.2. PARPi in Combination with CHK1i or ATRi Increases DNA Damage in BRCA^MUT^ and BRCA^WT^ Ovarian Cells

**Figure 3:**
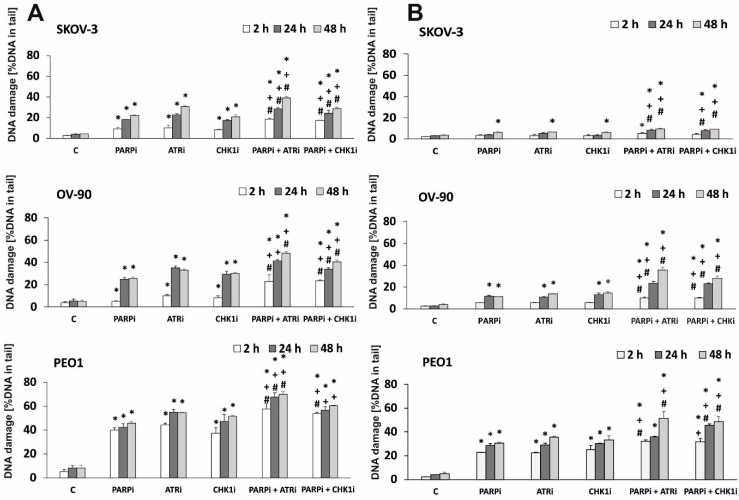
CHK1i and ATRi have a synergistic effect with PARPi on inducing DNA damage. The level of DNA damage in EOC cell lines treated with PARPi, ATRi, and CHK1i alone and in combination, measured as a percentage of the DNA in the comet tail. (**A**) The alkaline version, (**B**) the neutral version of the comet assay. Samples were treated for 2, 24, and 48 h. Error bars denote SD, * indicates statistically significant differences between the samples incubated with the drugs compared with control cells (*p* < 0.05). + Statistically significant differences between samples incubated with PARPi and combination treatment (PARPi/ATRi; PARPi/CHK1i) (*p* < 0.05). # Statistically significant differences between the samples incubated with ATRi or CHKi and combination treatment (PARPi/ATRi; PARPi/CHK1i) (*p* < 0.05). Statistical analysis was performed using the ANOVA test with the Tukey’s post-hoc test for multiple comparisons.

We hypothesized that treatment with the ATRi/CHK1i and PARPi combination may result in DNA damage. Alkali-labile sites, SSBs, and DSBs were analyzed by the comet assay, and the data are presented in Figure 3. The alkaline comet assay was performed after exposure to 0.5 µM of each drug for 2, 24, and 48 h. DNA damage in comet tails was detected as early as 2 h after treatment. The results of the comet assay showed that PARPi, ATRi, or CHK1i alone induced DNA damage in a time-dependent manner. The level of DNA damage was associated with cytotoxicity, measured by the MTT assay. DNA damage increased after PARPi treatment in BRCA^MUT^ and HR-proficient lines, and it increased markedly in HR-deficient cells (by approximately 1.8-fold). After 48 h of treatment, the level of DNA damage differed among the cell lines in response to ATRi (SKOV-3: 0.5%, OV-90: 33%, and PEO-1: 54.4%) or CHK1i (SKOV-3: 21%, OV-90: 30%, and PEO-1: 51%). Combination treatment with PARPi and ATRi increased the level of DNA damage in all cell lines after 24 h of incubation and the effect was not dependent on HR proficiency. Treatment with the combination of PARPi and ATRi caused a steady increase in DNA damage in comet tails that reached 38.9% in SKOV-3 cells, 48.2% in OV-90 cells, and 70.13% in PEO-1 cells after 48 h. In the OV-90 and PEO-1 cell lines, the effect of combination treatment on the level of DNA damage differed from that of PARPi alone by >15%.

We also tested the level of DNA damage using the neutral comet assay, which directly measures DNA DBSs. PARPi/ATRi and PARPi/CHK1i significantly increased DNA damage compared with each drug alone in a time-dependent manner (Figure 3B). Taken together, these results are consistent with the synergy observed in the antiproliferative studies. The representative images of comets are shown in Supplementary Figure S1.

### 2.3. Combined Treatment Induces Higher Levels of Apoptosis than Monotherapy in EOC Cells

**Figure 4:**
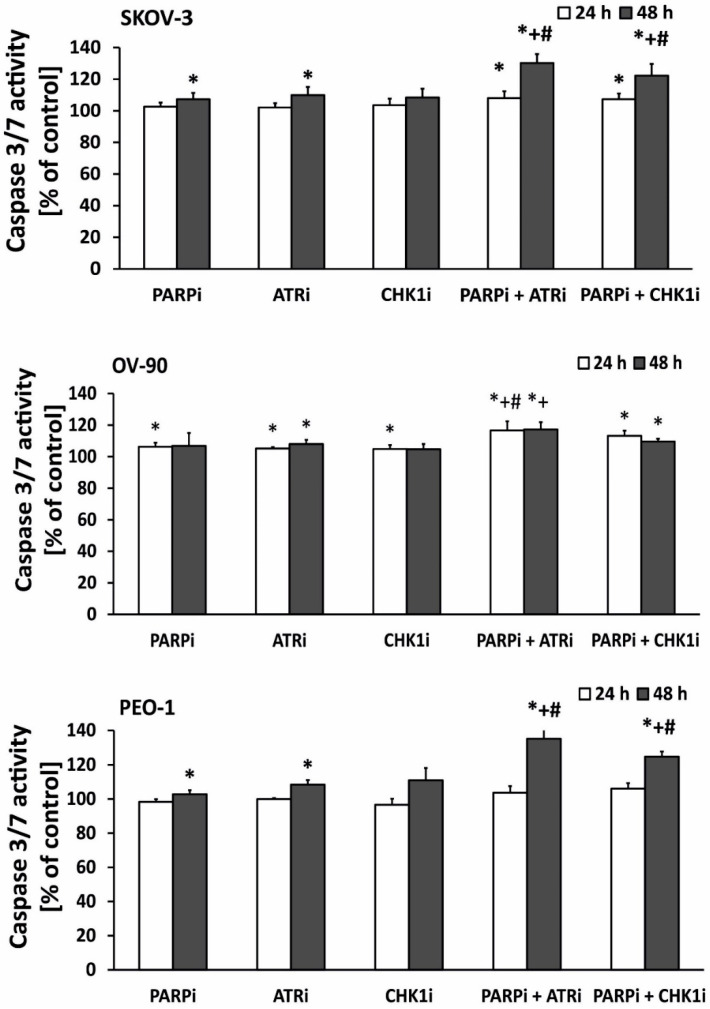
ATRi/CHK1i combined with PARPi increases apoptosis. Caspase 3/7 expression was used as an indicator of apoptosis in EOC cell lines exposed to 0.5 µmol/L PARPi, 0.5 µmol/L CHK1i, and 0.5 µmol/L ATRi for 24 and 48 h. Untreated cells were used as controls and considered 100%. In SKOV-3 cells, apoptosis was increased by both ATRi in combination with PARPi (30%) and CHK1 combination with PARPi (22%) after 48 h of treatment. In OV-90 (p53MUT) cells, apoptosis was increased after 24 and 48 h of incubation with ATRi in combination with PARPi (16% and 17%, respectively) and CHK1 in combination with PARPi (13% and 9% respectively). In PEO-1 (BRCA2^MUT^) cells, apoptosis was increased by ATRi in combination with PARPi (35%) and CHK1 in combination with PARPi (24%) after 48 h of treatment. Error bars denote standard deviation, * indicates statistically significant differences between the samples incubated with the drugs compared with control cells (*p* < 0.05). + Statistically significant differences between the samples incubated with PARPi and combination treatment (PARPi/ATRi; PARPi/CHK1i) (*p* < 0.05). # Statistically significant differences between samples incubated with ATRi or CHKi and combination treatment (PARPi/ATRi; PARPi/CHK1i). Statistical analysis was performed using the ANOVA test with the Tukey’s post-hoc test for multiple comparisons.

We showed that PARPi combined with ATRi and CHK1i increased DNA damage in EOC cell lines to a greater extent than PARPi, ATRi, and CHK1i alone. Because DNA damage induces apoptosis, we investigated the effect of the compounds on the caspase 3/7 ratio, a marker of apoptosis. The results are presented in Figure 4. Changes in the level of caspase 3/7 were time-dependent. Combination treatment induced apoptosis even in HR-proficient cells (SKOV-3 and OV-90 cells). PARPi, ATRi, or CHK1i alone at sub-lethal doses increased apoptosis only slightly. In all cell lines, the most significant changes in caspase activation were observed after 48 h of treatment. The PEO-1 cell line showed the highest levels of caspase 3/7 and was also the most sensitive cell line. After 48 h of treatment, ATRi combined with PARPi caused the highest increase in caspase 3/7 activity in all cell lines. For the CHK1i and PARPi combination, prolonged treatment time also caused a significant increase in caspase 3/7 activity. These results indicate that the major mechanism by which olaparib and ATRi/CHKi induced death of EOC cells may be apoptosis since we observed significant elevation of the caspase 3/7 activity. However, further studies are required to finally determine which processes are responsible for cell death.

## 3. Discussion

First-line chemotherapy for ovarian cancer, which is usually based on a platinum drug, has little impact on the overall survival of patients. Combination treatment with carboplatin and a taxane leads to a better overall clinical response than anthracyclines in patients with ovarian cancer [31,32]. However, the slight improvement in survival is associated with a significant increase in adverse effects, underscoring the need to identify and design novel therapeutic strategies.

The discovery of drugs that induce replication stress has emerged as a new research area to identify therapies against resistant tumors or tumors with poor prognosis. Olaparib, which is the most extensively studied PARPi, constitutes a significant step forward in ovarian cancer treatment. Phase II clinical trials revealed that it increases efficacy of the standard treatment when combined with carboplatin and paclitaxel. Progression-free survival was approximately 3 months longer in the olaparib with additional chemotherapy group than in the monotherapy group. Two additional PARP inhibitors have been approved by the FDA: rucaparib (Rubraca) and niraparib (Zejula), which are a promising class of agents for targeted EOC therapy [33]. The SOLO-1 Phase III trial demonstrated that olaparib therapy reduces the risk of disease progression or death by 70% in patients with newly diagnosed advanced BRCA-mutated ovarian cancer [13]. As a result, olaparib was approved as first-line maintenance therapy for BRCA^MUT^ advanced ovarian cancer. However, a small percentage (~15%) of EOC cases are BRCA-mutated. Most patients with high-grade serous EOC exhibit a phenotype of defective HR without BRCA mutations known as BRCAness [33]. One approach to overcome the limitations of olaparib is the use of cediranib, a vascular endothelial growth factor inhibitor. Cediranib suppresses pro-survival and anti-apoptotic signaling, thereby enhancing the efficacy of olaparib against BRCA^WT^ EOC cells [34]. Despite the contribution of olaparib to modern ovarian cancer therapy, the response rate in women is not satisfactory [35]. Identifying novel and effective treatments is extremely important. Therefore, the purpose of this study was to show that the efficacy of PARP inhibition can be increased by targeting critical cell cycle checkpoints, which may cause a significant reduction in the survival of EOC lines. Because the ATR/CHK1 pathway stabilizes replication forks and prevents DSBs [36], inhibition of ATR/CHK1 is expected to increase dependence on HR to complete replication. The combination of olaparib with anticancer agents that disrupt HR repair, namely, ATRi or CHK1i, represents a novel strategy to effectively sensitize ovarian cancer cells to olaparib.

We started our research by establishing cytotoxicity of drugs in a wide range of concentrations (0.1–30 µM) after 120 h of continuous incubation in order to make preliminary findings on their potential efficacy in cancer therapy. We showed that PARPi, ATRi, and CHK1i are cytotoxic in all the investigated ovarian cancer cell lines (SKOV-3, resistant, among others, to doxorubicin and cisplatin, grade 1/2, serous type (S); OV-90, human ovarian papillary serous adenocarcinoma, grade 3, high-grade serous (HGS); PEO-1, (BRCA2^MUT^; c.C4965G) grade 3). The results obtained by George et al. [37] are similar to our observations. They demonstrated that olaparib at 1 μM was significantly more cytotoxic to BRCA^MUT^ cell lines (PEO-1, JHOS4) than to the BRCA^REV^ (BRCA reverse) platinum-resistant PEO-4 cell line. Similar to PARPi, CHK1i was significantly more cytotoxic in BRCA2^MUT^ cells than in BRCA^WT^ ovarian cancer cells. On the other hand, ATRi (AZD6738) was cytotoxic to both BRCA^MUT^ and BRCA^WT^ cells [37]. In another study, it was shown that olaparib, after 5 days of treatment in the 0.1–1 µM range of concentration, was more cytotoxic in BRCA^MUT^ cells (PEO-1, JHOS4) compared with HR-proficient cells (PEO4^REV^, WO-20 ATR/CHK1 primary tumor cultures, BRCA^WT^). The authors also demonstrated that CHK1i (0.1–10 µM) was more effective in BRCA^MUT^ cells than in BRCA^WT^ cells as compared to ATRi (0.1–5 µM), which was equally active in all the tested cell lines [38].

We showed that ATRi and CHK1i increased the effect of olaparib in all the investigated ovarian cancer cell lines. To confirm the toxicity of the tested compounds and their combination, we performed a long-term survival assay (colony formation assay). The results were particularly significant in HR-deficient PEO-1 cells, in which combination therapy with ATRi decreased cell viability most effectively compared with olaparib alone. In HR-proficient lines (OV-90, SKOV-3), both combinations of inhibitors were also highly effective. Our results are in accordance with the study by Kim et al. [38] who revealed that the colony-forming ability after treatment with ATRi in combination with PARPi had been decreased. This observation confirmed a significantly higher cytotoxicity of the combination of ATRi and PARPi than the cytotoxicity of either drug alone in both BRCA2-deficient and wild-type cells [38]. The same group of authors extended cytotoxicity determination with multiple cell lines. Using Kuramochi (BRCA2^MUT^), FUOV1 (CCNE1-amplified; CCNE1^AMP^, Cyclin E gene amplification), OVKATE (BRCA^WT^ and CCNE1 copy normal), COV318 (CCNE1^AMP^), and OVCAR3 (CCNE1^AMP^), they reported findings similar to their previous study [39]. Moreover, ATR/CHK1 and DNA repair pathways were confirmed to be significantly altered in PARPi-resistant cells (PEO1-PR). ATRi alone was insufficient to overcome PARPi resistance in comparison to ATRi combined with PARPi [39]. Another study showed that the combination of PARPi with ATRi or CHK1i is as cytotoxic as the standard chemotherapy (carboplatin/paclitaxel) in BRCA^MUT^ cells (PEO-1, JHOS4) [37]. The combination of olaparib with both ATRi and CHK1i drastically decreases the viability of UWB-BRCA1^MUT^ ovarian cancer cells and UWB1-resistant cells compared with single agent administration [40]. Moreover, olaparib acts synergistically with ATRi (AZD6738) in ATM deficient cancer cells [41]. Combined treatment with ATRi and PARPi caused 84% cell death of ATM deficient cancer cells in contrast to only 37% growth inhibition in ATM-proficient wild-type cells [42]. The ATRi VE-821 sensitizes OVCAR-8, SKOV-3, and PEO-1 ovarian cancer cells to common chemotherapeutics, such as cisplatin, topotecan, and veliparib [26]. Furthermore, prexasertib (CHK1i) and olaparib monotherapy decrease cell viability in a dose-dependent manner in both BRCA^WT^ and BRCA^MUT^ ovarian cancer cells (OVCAR-3, OV-90, PEO-1, and PEO-4). Synergism was assessed at the lowest doses of prexasertib/olaparib (5 nM/5 μM) [43].

PARP1 coordinates and increases the speed of DNA repair, probably by detecting DNA lesions. The enzyme is essential not only for SSB repair binding, but also for DSBs, and is involved in the restart of stalled forks after release from replication blocks [44]. Small molecule inhibitors of PARP inhibit PARP activity, thereby preventing the dissociation of PARP from DNA damage sites on chromatin [45]. We detected a significant increase in PARP1 expression in all cell lines after exposure to olaparib. The combined action of the inhibitors led to a decrease in PARP expression in all cell lines. Osoegawa et al. showed that the basal level of PARP activity is positively correlated with PARP abundance in solid tumor cells [46], which is consistent with results of the present study. Other studies showed that PARP expression increases after exposure to 1.5 μM olaparib for 24 or 48 h. Studies on cervical cancer found a direct correlation between the abundance of PARP, its activity, and sensitivity to olaparib treatment. Cell lines with higher PARP expression are more sensitive to olaparib [47].

This study confirmed the link between PARP expression and the sensitivity to PARPi or inhibitors of replication stress proteins. DSBs and SSBs may result from the inactivation of tumor suppressor genes, activation of oncogenes, and alterations in the structure of mutator genes [48]. Aberrant products of ATR/CHK1 pathway genes may contribute to the induction, promotion, and progression of cancer transformation. DNA repair is the main cellular response to DNA damage. If cellular repair systems are ineffective against DNA damage, the repair time may be prolonged to allow for checkpoint activation [49]. PARPi increases ATR/CHK1 signaling and causes replication fork collapse into DSBs through different mechanisms. Thus, the combination of ATRi or CHK1i with PARPi would be more effective to diminish cancer cell survival. In the present study, we assessed the overall level of DNA damage, including SSBs and DSBs, using the alkaline and neutral variants of the comet assay. The results showed that ATRi and CHK1i had a marked effect on DNA strand breaks, inducing damage in all cell lines. Recently, the presence of DSBs was monitored by measuring the DSB marker γH2AX in BRCA2^MUT^ cells. The level of γH2AX increases in BRCA2^MUT^ cells relative to wild-type cells in the S-phase. The study suggests that fork destabilization or defects during the S-phase might be a source of DNA damage and leads to elevated sensitivity to combined action of PARPi and ATRi [38]. Prexasertib (CHK1i) predisposes cells to DSBs and allows cells to die [50]. ATR and CHK1 are pro-survival proteins playing a critical role in maintaining genome integrity against DNA damage. Synthetic lethality is a cellular phenomenon, indicating that simultaneous inactivation of two or more genes, which are nonlethal when inactivated alone, become lethal when inactivated together. Moreover, it is a consequence of the tendency to maintain genome stability despite various alterations occurring in the cell. Although BRCA^MUT^ tumors are selectively sensitive to PARPi [51], even a small number of persistent DSBs may be dangerous for the cancer cell. Simultaneous administration of ATRi/CHK1i and PARPi induced a higher level of DSBs than both compounds administered as single agents. Previously, it was described that PARPi/ATRi treatment exerts its dominant effects on replication by slowing fork progression and increasing fork asymmetry that leads to DSBs [39].

The highest level of DNA damage occurred in the BRCA^MUT^ cell line (PEO-1), although DNA damage was also observed in HR-proficient lines, such as SKOV-3 and OV-90 (HGSOCs) cells with p53MUT. The synergistic effect observed in our study was the result of high accumulation of DSBs after combination treatment with ATRi or CHK1i and olaparib in HR-deficient and HR-proficient cell lines. The increased genotoxic effect of the combined action of inhibitors may be related to a mutation in the p53 gene in the OV-90 line. The tumor suppressor p53 plays a crucial role in the DNA damage response. Mutation or loss of p53 occurs in approximately 50% of cancers [52]. However, whether p53 is a useful biomarker of the effect of RSR inhibitor treatment remains unclear. In this study, the OV-90 line was more sensitive to inhibitor treatment than the SKOV-3 line. Previous studies showed that checkpoint inhibitors synergize with genotoxins more effectively in cells with defects in the p53 pathway than in p53-proficient cells, which may be related to the increased replication stress caused by a relaxed S-phase entry [27,53]. Olaparib therapy combined with CHK1i selectively radiosensitizes p53 mutant pancreatic cancer cells, suggesting that inhibition of HR by CHK1i is a useful strategy for selective induction of a BRCA1 “deficient-like” phenotype in p53 mutant tumor cells sparing normal tissue [54].

The present results showing the effectiveness of the synergistic action of olaparib with the standard drug therapy or RSR modulators are consistent with previous studies. CDK4/6 inhibition by palbociclib has a synthetic lethal effect in combination with PARP inhibition by olaparib by inducing HR deficiency in a MYC-dependent manner in HGSOCs [55]. Combination treatment with BKM120 (phosphoinositide 3-kinase, PI3K, inhibitor) and olaparib is more effective than single agent treatment for inducing DNA damage in OVCAR-433, OVCAR-5, and OVCAR-8 cells, as indicated by the production of large numbers of DNA DSBs as a result of dual inhibition of PI3K and PARP. The combination of BKM120 and olaparib is effective in ovarian cancers with a broader spectrum of cancer-associated genetic alterations, but not limited to those with mutant PI3KCA or BRCA genes [56]. Exposure of cells to PARPi PJ34 blocks CHK1i-induced PARP1 activation and PARP1 adenosine diphosphate (ADP) ribosylation [57]. Suppression of ATR by VE-821 (ATRi) can affect HR repair in ovarian cancer cells, leading to accumulation of DNA DSBs in cell nuclei, thereby increasing the sensitivity of ovarian cancer SKOV-3 cells to cisplatin [58]. Therefore, inhibition of ATR/CHK1 and PARP together increases DSB generation from fork collapse and diminished alternative DNA repair mechanisms.

Cells treated with replication stress inhibitors die through two major pathways: apoptosis (the predominant type of programmed cell death) or necrosis. Both processes differ biochemically and morphologically. Cell destruction through the apoptotic pathway is the most effective way to eradicate cancer cells because it does not cause inflammation in adjacent healthy tissues. Programmed cell death ensures removal of mutated, infected, or damaged cells and maintains normal tissue homeostasis, regulating the balance between cell division and a “silent disappearance” of cells. The proliferation and colony formation assays used in this study did not clarify whether the toxic effects of the investigated drugs resulted from the activation of the apoptotic cell death machinery. PARPi, as well as ATRi and CHK1i, also cause necrotic cell death. Therefore, we examined the type of cell death induced by the drugs and the role of apoptosis in their cytotoxic and antiproliferative activities. Members of the caspase family are crucial mediators of the complex biochemical events associated with apoptosis. Caspase 3, a key effector in the apoptotic pathway, amplifies the signal from initiator caspases leading to full commitment to cellular disassembly. It was demonstrated that PARPi/ATRi combination treatment significantly increased apoptosis compared with monotherapy in HR-deficient and HR-proficient ovarian cancer cells. Our results are in agreement with the study performed on BRCA^MUT^ PEO-1 and BRCA^REV^ PEO-4 cell lines. Cleaved caspase 3 after 48 h of treatment with 1 µM CHK1i and 1 µM ATRi was significantly increased as compared with untreated control cells. However, contrary to our results, the combination of PARPi/ATRi and PARPi/CHK1i did not substantially increase caspase 3 in relation to each agent alone [38]. Interestingly, the combination of drugs led to worse apoptosis than monotherapy in the olaparib and carboplatin-resistant cell lines (PEO1-PR, JHOS4-PR, PEO1-CR, OVCAR-3) [39]. Additional studies are necessary to confirm whether apoptosis is a leading process responsible for cytotoxicity of tested inhibitors’ combinations.

The presented results indicate that ovarian cells harboring loss-of-function mutations other than BRCA may also benefit from novel treatments. In addition, most EOC patients are BRCA^WT^, underscoring the importance of these results. We also showed that the combination of ATRi and PARPi is more cytotoxic than that of CHK1i and PARPi. Thus, ATR may stabilize replication forks independently from CHK1 [59]. Our observation can also be explained by the action of MRE11 that mediates PARPi sensitivity of BRCA1-deficient cells. ATRi disrupts BRCA1-independent RAD51 leading to DSBs and stalled forks in PARPi-resistant BRCA1-deficient cells. Thus, ATRi enables MRE11-mediated fork degradation [60]. Another study also confirmed that ATR plays the main role in avoiding replication catastrophe. Two- to ten-fold reduction in fork speed led to global chromatin recruitment of sensors and mediators of the ATR pathway without substantial activation of CHK1, ATM, or p53. Analysis of the cell phenotypes depleted of ATR or CHK1 and their exposure to moderate levels of stress shows that ATR, but not CHK1, is crucial for common fragile site (CFS) integrity. Moreover, in ATR-deficient cells, single stranded DNA (ssDNA) foci result from the MRE11-dependent resection of collapsed forks, suggesting that long stretches of ssDNA are a consequence rather than a cause of CFS instability [61]. It is also known that RPA protein (replication protein A) binds to single-stranded DNA generated in the replication forks. The study by Toledo et al. confirmed that if replication is blocked, ssDNA is formed, linked to RPA, and led to the recruitment of the ATR kinase. Although ATR requires the RPA-coated ssDNA for its own activation, the endpoint of ATR signaling to protect forks against fatal breakage also feeds back on the level of RPA. Partial reduction in RPA enhanced fork breakage and forced elevation of RPA was sufficient to delay such replication catastrophe even in the absence of ATR activity. ATR inhibition increases ssDNA break generation up to the point where it would deplete all available RPA [62]. The research by Mengwasser et al. also points to the extremely important role of ATR kinases. They found that BRCA2^MUT^ cells (colonic DLD-1 BRCA2^MUT^ cell line and PEO-1 BRCA2^MUT^) are dependent on ATR activation; that was confirmed by using VE-821 as ATRi. Ape2 (Apurinic/apyrimidinic endonuclease) depletion led to a more prominent phosphorylation of CHK1 and RPA32 in the BRCA2^MUT^ cell line than in the BRCA2^WT^ cell line (C4–2 clone that contains BRCA^REV^ selected for cisplatin resistance), suggesting increased ATR activation [63].

Although the efficacy of the PARPi/ATRi and PARPi/CHK1i combinations differed, each of these combinations was significantly more effective than single agent treatment in ovarian cancer cells.

## 4. Materials and Methods

### 4.1. Reagents

Culture media (RPMI 1640, DMEM) were obtained from Gibco (Thermo Fisher Scientific, Waltham, MA, USA), and fetal bovine serum (FBS) was from Capicorn Scientific GmbH (Ebsdorfergrund, Germany); trypsin–EDTA, penicillin, and streptomycin were acquired from Sigma-Aldrich (St. Louis, MO, USA). CellEvent™ Caspase-3/7 Green Detection Reagent was from Thermo Fisher Scientific, Waltham, MA, USA (cat # C10423). PARPi (AZD2281), ATRi (AZD6738), and CHK1i (MK8776) were purchased from Selleckchem (Munich, Germany). Other chemicals and solvents were of high analytical grade and were obtained from Sigma-Aldrich or Avantor Performance Materials Poland S.A. (Gliwice, Poland).

### 4.2. Cell Culture and Drug Administration

The human OV-90 (human malignant papillary serous carcinoma, American Type Culture Collection (ATCC) CRL-11732™) and SKOV-3 (human ovarian adenocarcinoma, ATCC HTB-77) cell lines were bought from ATCC (Rockville, MD, USA), and PEO-1 cells (human ovarian cancer; estrogen rec, 10032308) for preliminary research were donated by Dr. Gallo (Fondazione Policlinico Universitario A. Gemelli, IRCCS, Rome, Italy), and then bought from the European Collection of Authenticated Cell Cultures. The newly received cells were expanded and aliquots of less than 10 passages were stored in liquid nitrogen. All cell lines were kept at low passage, returning to original frozen stocks every 6 months. During the course of the study, cells were thawed and passaged within 2 months in each experiment. The cells were cultured in DMEM and RPMI with 10% FBS, penicillin (10 U/mL), and streptomycin (50 µg/mL) and regularly checked for mycoplasma contamination. The cells were cultured in an atmosphere of 5% CO2 and 95% air at 37 °C.

### 4.3. MTT Assay

To select the most effective PARPi/ATRi and PARPi/CHK1i ratios, several concentration ratios of the compounds (1:1; 2:1; 10:1; 1:2; 1:10) were tested at the lowest effective concentration based on the survival curve (0.5 µM). To analyze the drug interaction between ATRi and CHK1i and PARPi combined with either agent, the coefficient of drug interaction (CDI) was calculated [64]. The CDI is defined by the following formula: CDI = AB/(A × B). According to the absorbance of each group, AB is the ratio of the two-drug combination group to the untreated control group, and A or B is the ratio of the single drug group to the control group. A CDI < 1 indicates synergism, CDI < 0.7—significant synergism, CDI = 1—additivity, and CDI > 1—antagonism.

### 4.4. Clonogenic Assay

The effect of PARPi, CHK1i, and ATRi on cell growth was assessed using a clonogenic assay. For this analysis, 200 cells were plated onto six-well plates in a growth medium, and after overnight attachment, cells were exposed to the test compounds for 5 days. The cells were washed with a fresh medium and allowed to grow for 10–14 days under drug-free conditions. Then, the cell colonies were fixed with methanol mixed with acetic acid (7:1) for 10 min and stained with 0.5% crystal violet for 20 min. The plates were rinsed with water, air-dried, photographed, and evaluated for colony estimation. Colonies containing more than 50 cells were counted. For each sample, the results from three replicates were averaged.

### 4.5. Morphological Assessment

SKOV-3, OV-90, and PEO-1 cells were treated with olaparib, ATRi, CHK1i, and a combination of PARPi with ATRi or PARPi with CHK1i, each at a concentration of 0.5 µM, for 24 h and then examined under an inverted optical microscope (Olympus IX70, Tokyo, Japan).

### 4.6. Western Blot Analysis

The cells were lysed in a cell extraction buffer (Invitrogen™, Waltham, MA, USA) containing a protease inhibitor cocktail and phenylmethylsulfonyl fluoride (PMSF) (Roche, Mannheim, Germany) in accordance with the manufacturer’s protocol. The protein concentration was determined using the Bradford method. SDS polyacrylamide gel electrophoresis and the wet transfer of proteins (35 µg per lane) onto 0.45 µm polyvinylidene difluoride (PVDF) membranes were performed using standard procedures. To confirm equal loading and transfer, Precision Plus Protein™ WesternC™ Blotting Standards were used. After blocking nonspecific sites using 5% bovine serum albumin (BSA) (Sigma-Aldrich) or 5% non-fat dry milk, membranes were incubated with the rabbit monoclonal antibody against PARP at a dilution of 1/1000 (cat. # 9532), phospho-ATR (Thr1989) (cat. # 30632), total ATR (cat. # 13934), phospho-CHK1 (Ser345) (cat. # 2348), and glyceraldehyde 3-phosphate dehydrogenase (GAPDH) (cat. #2118), all from Cell Signaling Technology, Inc. (Danvers, MA, USA), and the rabbit anti-CHK1 polyclonal antibody (cat. # STJ92269, St John’s Laboratory). The membranes were then exposed to the anti-rabbit IgG horseradish peroxidase-conjugated secondary antibody (cat. # 7074, Cell Signaling Technology), followed by detection using a chemiluminescent substrate (Thermo Fisher Scientific, Waltham, MA, USA). Immunoreactive bands were visualized using a DNR MicroChemi system. Band intensities were quantified using the ImageJ software (NIH, Bethesda, MD, USA). The integrated optical density of the bands was measured in a digitized image. Relative expression was expressed as the ratio of the densitometric volume of the test band to that of the respective GAPDH band.

### 4.7. Comet Assay

The alkaline version of single cell gel electrophoresis (comet assay) was used to detect alkali-labile sites, single-strand breaks (SSBs), and DSBs caused by exposure to the tested compounds. Each experiment was repeated three times. In the neutral versions, the tail DNA percentage is positively correlated with DSBs. The cells were plated on 12-well plates (105 cells/well) and treated with PARPi, ATRi, CHKi, and their combination at the 0.5 µM dose for 2, 24, and 48 h at 37 °C. Next, the cells were collected into Eppendorf tubes and rinsed with PBS. The cells were then suspended in 0.75% low melting point agarose dissolved in PBS (pH 7.4) and placed on microscope slides precoated with 0.5% normal melting point agarose. Subsequently, the slides were treated with a cooled lysis buffer (2.5 M NaCl, 100 mM EDTA, 10 mM Tris, 1% Triton X-100, pH 9.0) for 1–24 h at 4 °C. Then, slides were placed in the developing buffer (300 mM NaOH, 1 mM EDTA) for 20 min. Electrophoresis was performed in a buffer composed of 30 mM NaOH and 1 mM EDTA at 0.73 V/cm and 290 mA for 20 min. In the neutral version of the comet assay, electrophoresis was run in a buffer consisting of 100 mM Tris and 300 mM sodium acetate, with the pH adjusted to 9.0 using glacial acetic acid. Electrophoresis was performed for 60 min after a 20 min equilibrium period at an electric field strength of 0.41 V/cm and 50 mA at 4 °C.

The samples were stained with 4,6-diamidino-2-phenylindole (DAPI) (1 g/mL). The slides were stored in a wet chamber at 4 °C and analyzed under a fluorescence microscope. Fifty randomly selected cells from each slide were measured using an image analysis system (Nikon, Japan) attached to a COHU 4910 video camera, which was equipped with a UV-1 filter block consisting of an excitation filter (359 nm) and a barrier filter (461 nm) connected to the image analysis system Lucia-Comet v. 4.51 (Laboratory Imaging, Prague, Czech Republic) [30].

### 4.8. Caspase 3/7 Assay

The activities of caspases 3 and 7 were estimated with CellEvent™ Caspse-3/7 Green Detection Reagent (Thermo Fisher Scientific, Waltham, MA, USA) according to the manufacturer’s protocol. The cells were seeded on 96-well plates (15 × 103/well) and after 24 h they were incubated with the appropriate drugs for 24 h or 48 h. The cells were fixed by adding a final concentration of 4% paraformaldehyde solution (10 min, room temperature). Cells were labeled with CellEvent™ Caspase-3/7 Green Detection Reagent (5 µM) diluted in PBS with 5% FBS to avoid fluorescence background. After activation of caspase 3/7 in apoptotic cells, the four amino acid (Asp-Glu-Val-Asp, DEVD) peptide was cleaved, enabling the dye to bind to DNA, which produced a bright fluorogenic response with absorption/emission maxima of 502/530 nm according to the manufacturer’s protocol. Fluorescence intensity was measured using a Fluoroskan Ascent FL plate reader (Labsystem, Stockholm, Sweden). Cysteine protease activity was expressed as a ratio of the fluorescence of drug-treated samples to that of the corresponding untreated controls (taken as 100%).

### 4.9. Statistical Analysis

The data are presented as means ± SD of at least three independent experiments. Statistical analyses were performed with the Student’s t-test and ANOVA with the Tukey’s post-hoc test for multiple comparisons as appropriate (StatSoft, Tulsa, OK, USA) [30]. *p*-values < 0.05 were considered statistically significant.

## 5. Conclusions

**Figure 5:**
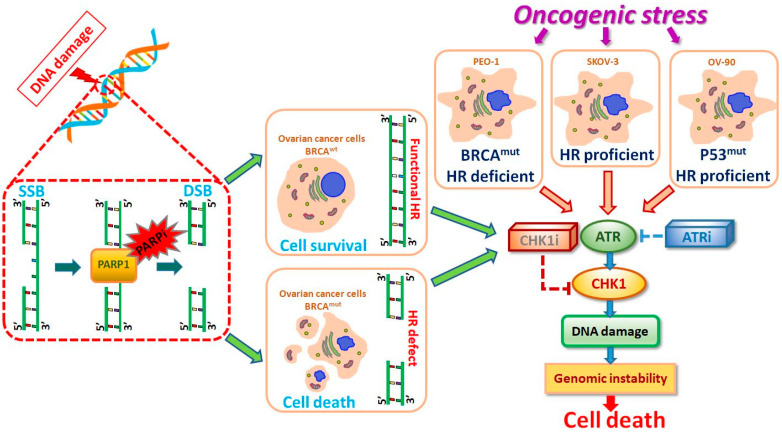
Proposed model of the molecular and cellular responses to new replication stress inhibitors. ATR/CHK1 stabilizes replication forks and prevents their collapse into DSBs. SSBs can be accurately repaired using the undamaged strand as a template, a process involving the PARP enzyme. SSBs are mainly repaired through the homologous recombination (HR) pathway. BRCA is related to the error-free repair of DSBs by HR. ATRi or CHK1i in monotherapy and in combined treatment with PARPi cause genome instability and leads to the synthetic lethality of ovarian cancer cells.

ATR and CHK1 suppressed DNA break formation and induced DSB repair to remove DNA damage (Figure 5). PARPi increased the dependence on ATR/CHK1 for maintaining genome stability. Combination treatment with PARPi and ATRi completely suppressed ovarian cell growth independently from HR effectiveness. Inhibition of ATR or CHK1 concomitant with PARP inhibition increased DSB generation, resulting in apoptosis induction. The present results were obtained at sub-lethal doses, underscoring the high potential of the inhibitors tested. The effect of PARPi/ATRi and PARPi/CHK1i was dependent on the genetics of the tumor. The present results confirmed the sensitivity of ovarian cancer cells to CHK1i and especially to ATRi. The simultaneous use of olaparib with inhibitors of RSR proteins leads to synthetic lethality and sensitizes cells to olaparib therapy by blocking DNA repair. Thus, the present data may provide new prospects for the treatment of ovarian cancer patients and establish a basis for further research.

## Supplementary Materials

The following are available online at https://www.mdpi.com/1422-0067/21/24/9715/s1.

## Abbreviations

ADP	Adenosine diphosphate
ATM	Ataxia telangiectasia mutated protein
ATR	Ataxia telangiectasia and Rad3-related protein
ATRi	Ataxia telangiectasia and Rad3-related protein inhibitor
BSA	Bovine serum albumin
CDI	Coefficient of drug interaction
CHK1	Checkpoint kinase 1
CHK1i	Checkpoint kinase 1 inhibitor
DAPI	4,6-diamidino-2-phenylindole
DSB	Double-strand break
FANCI	Fanconi anemia complementation group I
GAPDH	Glyceraldehyde 3-phosphate dehydrogenase
HRR	Homologous recombination repair
MCM2	DNA replication licensing factor
MTT	3-(4,5-Dimethylthiazol-2-yl)-2,5-Diphenyltetrazolium Bromide
PARP	Poly (ADP-ribose) polymerase
PARPi	Poly (ADP-ribose) polymerase inhibitor
PMSF	Phenylmethylsulfonyl fluoride
PVDF	Polyvinylidene difluoride
RPA	Replication protein A
SMARCAL1	SWI/SNF-related matrix-associated actin-dependent regulator of chromatin subfamily A-like protein 1
SSB	Single-strand break
ssDNA	single stranded DNA
WRN	Werner syndrome ATP-dependent helicase

## References

[1] He S., Zhang C., Liu L., Li L., Duan Y., Liu J., Shao F. (2020). Efficacy and safety of PARP inhibitors as the maintenance therapy in ovarian cancer: A meta-analysis of nine randomized controlled trials. Biosci. Rep..

[2] (2011). Integrated genomic analyses of ovarian carcinoma. Nature.

[3] Buttarelli M., De Donato M., Raspaglio G., Babini G., Ciucci A., Martinelli E., Baccaro P., Pasciuto T., Fagotti A., Scambia G. (2020). Clinical Value of lncRNA MEG3 in High-Grade Serous Ovarian Cancer. Cancers.

[4] Konstantinopoulos P.A., Ceccaldi R., Shapiro G.I., D’Andrea A.D. (2015). Homologous Recombination Deficiency: Exploiting the Fundamental Vulnerability of Ovarian Cancer. Cancer Discov..

[5] Choi M.C., Hwang S., Kim S., Jung S.G., Park H., Joo W.D., Song S.H., Lee C., Kim T.-H., Kang H. (2020). Clinical Impact of Somatic Variants in Homologous Recombination Repair-Related Genes in Ovarian High-Grade Serous Carcinoma. Cancer Res. Treat..

[6] Martí J.M., Fernández-Cortés M., Serrano-Sáenz S., Zamudio-Martinez E., Delgado-Bellido D., Garcia-Diaz A., Oliver F.J. (2020). The Multifactorial Role of PARP-1 in Tumor Microenvironment. Cancers.

[7] Bohrer R.C., Dicks N., Gutierrez K., Duggavathi R., Bordignon V. (2018). Double-strand DNA breaks are mainly repaired by the homologous recombination pathway in early developing swine embryos. FASEB J..

[8] Spriggs D.R., Longo D.L. (2018). Progress in BRCA-Mutated Ovarian Cancer. N. Engl. J. Med..

[9] Sánchez H., Paul M.W., Grosbart M., Van Rossum-Fikkert S.E., Lebbink J.H.G., Kanaar R., Houtsmuller A.B., Wyman C. (2017). Architectural plasticity of human BRCA2–RAD51 complexes in DNA break repair. Nucleic Acids Res..

[10] Rajawat J., Shukla N., Mishra D.P. (2017). Therapeutic Targeting of Poly(ADP-Ribose) Polymerase-1 (PARP1) in Cancer: Current Developments, Therapeutic Strategies, and Future Opportunities. Med. Res. Rev..

[11] Tinker A.V., Gelmon K. (2012). The role of PARP inhibitors in the treatment of ovarian carcinomas. Curr. Pharm. Des..

[12] Kim G., Ison G., McKee A.E., Zhang H., Tang S., Gwise T., Sridhara R., Lee E., Tzou A., Philip R. (2015). FDA Approval Summary: Olaparib Monotherapy in Patients with Deleterious Germline BRCA-Mutated Advanced Ovarian Cancer Treated with Three or More Lines of Chemotherapy. Clin. Cancer Res..

[13] Moore K., Colombo N., Scambia G., Kim B.-G., Oaknin A., Friedlander M., Lisyanskaya A., Floquet A., Leary A., Sonke G.S. (2018). Maintenance Olaparib in Patients with Newly Diagnosed Advanced Ovarian Cancer. N. Engl. J. Med..

[14] Consortium A.P.G. (2017). AACR Project GENIE: Powering Precision Medicine through an International Consortium. Cancer Discov..

[15] Pettitt S.J., Krastev D.B., Brandsma I., Drean A., Song F., Aleksandrov R., Harrell M.I., Menon M., Brough R., Campbell J. (2018). Genome-wide and high-density CRISPR-Cas9 screens identify point mutations in PARP1 causing PARP inhibitor resistance. Nat. Commun..

[16] Montemorano L., Lightfoot M., Bixel K. (2019). Role of Olaparib as Maintenance Treatment for Ovarian Cancer: The Evidence to Date. OncoTargets Ther..

[17] Engelke C.G., Parsels L.A., Qian Y., Zhang Q., Karnak D., Robertson J.R., Tanska D.M., Wei D., Davis M.A., Parsels J.D. (2013). Sensitization of Pancreatic Cancer to Chemoradiation by the Chk1 Inhibitor MK8776. Clin. Cancer Res..

[18] Gupta D., Lin B., Cowan A., Heinen C.D. (2018). ATR-Chk1 activation mitigates replication stress caused by mismatch repair-dependent processing of DNA damage. Proc. Natl. Acad. Sci. USA.

[19] Menolfi D., Zha S. (2020). ATM, ATR and DNA-PKcs kinases—The lessons from the mouse models: Inhibition ≠ deletion. Cell Biosci..

[20] Saldivar J.C., Cortez D., Cimprich K.A. (2017). The essential kinase ATR: Ensuring faithful duplication of a challenging genome. Nat. Rev. Mol. Cell Biol..

[21] Zhang X., Lu X., Akhter S., Georgescu M.-M., Legerski R.J. (2016). FANCI is a negative regulator of Akt activation. Cell Cycle.

[22] Yeom G., Kim J., Park C.-J. (2019). Investigation of the core binding regions of human Werner syndrome and Fanconi anemia group J helicases on replication protein A. Sci. Rep..

[23] Pugliese G.M., Salaris F., Palermo V., Marabitti V., Morina N., Rosa A., Franchitto A., Pichierri P. (2019). Inducible SMARCAL1 knockdown in iPSC reveals a link between replication stress and altered expression of master differentiation genes. Dis. Models Mech..

[24] Kim H.-J., Min A., Im S.-A., Jang H., Lee K.H., Lau A., Lee M., Kim S., Yang Y., Kim J. (2017). Anti-tumor activity of the ATR inhibitor AZD6738 in HER2 positive breast cancer cells. Int. J. Cancer.

[25] Gamper A.M., Rofougaran R., Watkins S.C., Greenberger J.S., Beumer J.H., Bakkenist C.J. (2013). ATR kinase activation in G1 phase facilitates the repair of ionizing radiation-induced DNA damage. Nucleic Acids Res..

[26] Huntoon C.J., Flatten K.S., Wahner Hendrickson A.E., Huehls A.M., Sutor S.L., Kaufmann S.H., Karnitz L.M. (2013). ATR Inhibition Broadly Sensitizes Ovarian Cancer Cells to Chemotherapy Independent of BRCA Status. Cancer Res..

[27] Reaper P.M., Griffiths M.R., Long J.M., Charrier J.-D., MacCormick S., Charlton P.A., Golec J.M.C., Pollard J.R. (2011). Selective killing of ATM- or p53-deficient cancer cells through inhibition of ATR. Nat. Chem. Biol..

[28] Kawahara N., Ogawa K., Nagayasu M., Kimura M., Sasaki Y., Kobayashi H. (2017). Candidate synthetic lethality partners to PARP inhibitors in the treatment of ovarian clear cell cancer. Biomed. Rep..

[29] Ciardo D., Goldar A., Marheineke K. (2019). On the Interplay of the DNA Replication Program and the Intra-S Phase Checkpoint Pathway. Genes.

[30] Rogalska A., Gajek A., Marczak A. (2014). Epothilone B induces extrinsic pathway of apoptosis in human SKOV-3 ovarian cancer cells. Toxicol. Vitr..

[31] Kampan N.C., Madondo M.T., McNally O.M., Quinn M., Plebanski M. (2015). Paclitaxel and Its Evolving Role in the Management of Ovarian Cancer. BioMed Res. Int..

[32] De Donato M., Righino B., Filippetti F., Battaglia A., Petrillo M., Pirolli D., Scambia G., De Rosa M.C., Gallo D. (2018). Identification and antitumor activity of a novel inhibitor of the NIMA-related kinase NEK6. Sci. Rep..

[33] Ahmad A., Lin Z.P., Zhu Y.-L., Lo Y.-C., Moscarelli J., Xiong A., Korayem Y., Huang P.H., Giri S., LoRusso P. (2018). Combination of triapine, olaparib, and cediranib suppresses progression of BRCA-wild type and PARP inhibitor-resistant epithelial ovarian cancer. PLoS ONE.

[34] Hjortkjær M., Malik Aagaard Jørgensen M., Waldstrøm M., Ørnskov D., Søgaard-Andersen E., Jakobsen A., Dahl-Steffensen K. (2019). The clinical importance of BRCAness in a population-based cohort of Danish epithelial ovarian cancer. Int. J. Gynecol. Cancer.

[35] Baloch T., López-Ozuna V.M., Wang Q., Matanis E., Kessous R., Kogan L., Yasmeen A., Gotlieb W.H. (2019). Sequential therapeutic targeting of ovarian Cancer harboring dysfunctional BRCA1. BMC Cancer.

[36] Kim W., Zhao F., Wu R., Qin S., Nowsheen S., Huang J., Zhou Q., Chen Y., Deng M., Guo G. (2019). ZFP161 regulates replication fork stability and maintenance of genomic stability by recruiting the ATR/ATRIP complex. Nat. Commun..

[37] George E., Kim H., Krepler C., Wenz B., Makvandi M., Tanyi J.L., Brown E., Zhang R., Brafford P., Jean S. (2017). A patient-derived-xenograft platform to study BRCA-deficient ovarian cancers. JCI Insight.

[38] Kim H., George E., Ragland R.L., Rafail S., Zhang R., Krepler C., Morgan M.A., Herlyn M., Brown E.J., Simpkins F. (2017). Targeting the ATR/CHK1 Axis with PARP Inhibition Results in Tumor Regression in BRCA-Mutant Ovarian Cancer Models. Clin. Cancer Res..

[39] Kim H., Xu H., George E., Hallberg D., Kumar S., Jagannathan V., Medvedev S., Kinose Y., Devins K., Verma P. (2020). Combining PARP with ATR inhibition overcomes PARP inhibitor and platinum resistance in ovarian cancer models. Nat. Commun..

[40] Burgess B.T., Anderson A.M., McCorkle J.R., Wu J., Ueland F.R., Kolesar J.M. (2020). Olaparib Combined with an ATR or Chk1 Inhibitor as a Treatment Strategy for Acquired Olaparib-Resistant BRCA1 Mutant Ovarian Cells. Diagnostics.

[41] Jette N.R., Kumar M., Radhamani S., Arthur G., Goutam S., Yip S., Kolinsky M., Williams G.J., Bose P., Lees-Miller S.P. (2020). ATM-Deficient Cancers Provide New Opportunities for Precision Oncology. Cancers.

[42] Lloyd R., Falenta K., Wijnhoven P.W., Chabbert C., Stott J., Yates J., Lau A.Y., Young L.A., Hollingsworth S.J. Abstract 337: The PARP inhibitor olaparib is synergistic with the ATR inhibitor AZD6738 in ATM deficient cancer cells. Proceedings of the Molecular and Cellular Biology/Genetics, American Association for Cancer Research (AACR).

[43] Brill E., Yokoyama T., Nair J., Yu M., Ahn Y.-R., Lee J.-M. (2017). Prexasertib, a cell cycle checkpoint kinases 1 and 2 inhibitor, increases in vitro toxicity of PARP inhibition by preventing Rad51 foci formation in BRCA wild type high-grade serous ovarian cancer. Oncotarget.

[44] Ray Chaudhuri A., Nussenzweig A. (2017). The multifaceted roles of PARP1 in DNA repair and chromatin remodelling. Nat. Rev. Mol. Cell Biol..

[45] Parsels L.A., Karnak D., Parsels J.D., Zhang Q., Vélez-Padilla J., Reichert Z.R., Wahl D.R., Maybaum J., O’Connor M.J., Lawrence T.S. (2018). PARP1 Trapping and DNA Replication Stress Enhance Radiosensitization with Combined WEE1 and PARP Inhibitors. Mol. Cancer Res..

[46] Osoegawa A., Gills J.J., Kawabata S., Dennis P.A. (2017). Rapamycin sensitizes cancer cells to growth inhibition by the PARP inhibitor olaparib. Oncotarget.

[47] Bianchi A., Lopez S., Altwerger G., Bellone S., Bonazzoli E., Zammataro L., Manzano A., Manara P., Perrone E., Zeybek B. (2019). PARP-1 activity (PAR) determines the sensitivity of cervical cancer to olaparib. Gynecol. Oncol..

[48] Parmar K., Kochupurakkal B.S., Lazaro J.-B., Wang Z.C., Palakurthi S., Kirschmeier P.T., Yang C., Sambel L.A., Färkkilä A., Reznichenko E. (2019). The CHK1 Inhibitor Prexasertib Exhibits Monotherapy Activity in High-Grade Serous Ovarian Cancer Models and Sensitizes to PARP Inhibition. Clin. Cancer Res..

[49] Szczepanska J., Poplawski T., Synowiec E., Pawlowska E., Chojnacki C.J., Chojnacki J., Blasiak J. (2011). 2-Hydroxylethyl methacrylate (HEMA), a tooth restoration component, exerts its genotoxic effects in human gingival fibroblasts trough methacrylic acid, an immediate product of its degradation. Mol. Biol. Rep..

[50] Angius G., Tomao S., Stati V., Vici P., Bianco V., Tomao F. (2019). Prexasertib, a checkpoint kinase inhibitor: From preclinical data to clinical development. Cancer Chemother. Pharmacol..

[51] Liu Y., Burness M.L., Martin-Trevino R., Guy J., Bai S., Harouaka R., Brooks M.D., Shang L., Fox A., Luther T.K. (2017). RAD51 Mediates Resistance of Cancer Stem Cells to PARP Inhibition in Triple-Negative Breast Cancer. Clin. Cancer Res..

[52] Baugh E.H., Ke H., Levine A.J., Bonneau R.A., Chan C.S. (2017). Why are there hotspot mutations in the TP53 gene in human cancers?. Cell Death Differ..

[53] Karnitz L.M., Zou L. (2015). Molecular Pathways: Targeting ATR in Cancer Therapy. Clin. Cancer Res..

[54] Vance S., Liu E., Zhao L., Parsels J.D., Parsels L.A., Brown J.L., Maybaum J., Lawrence T.S., Morgan M.A. (2014). Selective radiosensitization of p53 mutant pancreatic cancer cells by combined inhibition of Chk1 and PARP1. Cell Cycle.

[55] Yi J., Liu C., Tao Z., Wang M., Jia Y., Sang X., Shen L., Xue Y., Jiang K., Luo F. (2019). MYC status as a determinant of synergistic response to Olaparib and Palbociclib in ovarian cancer. EBioMedicine.

[56] Wang D., Li C., Zhang Y., Wang M., Jiang N., Xiang L., Li T., Roberts T.M., Zhao J.J., Cheng H. (2016). Combined inhibition of PI3K and PARP is effective in the treatment of ovarian cancer cells with wild-type PIK3CA genes. Gynecol. Oncol..

[57] Mitchell C., Park M., Eulitt P., Yang C., Yacoub A., Dent P. (2010). Poly(ADP-Ribose) Polymerase 1 Modulates the Lethality of CHK1 Inhibitors in Carcinoma Cells. Mol. Pharmacol..

[58] Zhou J., Yang F., Zhou L., Wang J.-g., Wen P., Luo H., Li W., Song Z., Sharman E.H., Bondy S.C. (2014). Dietary melatonin attenuates age-related changes in morphology and in levels of key proteins in globus pallidus of mouse brain. Brain Res..

[59] Stingele J., Bellelli R., Alte F., Hewitt G., Sarek G., Maslen S.L., Tsutakawa S.E., Borg A., Kjær S., Tainer J.A. (2016). Mechanism and Regulation of DNA-Protein Crosslink Repair by the DNA-Dependent Metalloprotease SPRTN. Mol. Cell.

[60] Yazinski S.A., Comaills V., Buisson R., Genois M.M., Nguyen H.D., Ho C.K., Todorova Kwan T., Morris R., Lauffer S., Nussenzweig A. (2017). ATR inhibition disrupts rewired homologous recombination and fork protection pathways in PARP inhibitor-resistant BRCA-deficient cancer cells. Genes Dev..

[61] Koundrioukoff S., Carignon S., Techer H., Letessier A., Brison O., Debatisse M. (2013). Stepwise activation of the ATR signaling pathway upon increasing replication stress impacts fragile site integrity. PLoS Genet..

[62] Toledo L.I., Altmeyer M., Rask M.B., Lukas C., Larsen D.H., Povlsen L.K., Bekker-Jensen S., Mailand N., Bartek J., Lukas J. (2013). ATR prohibits replication catastrophe by preventing global exhaustion of RPA. Cell.

[63] Mengwasser K.E., Adeyemi R.O., Leng Y., Choi M.Y., Clairmont C., D’Andrea A.D., Elledge S.J. (2019). Genetic Screens Reveal FEN1 and APEX2 as BRCA2 Synthetic Lethal Targets. Mol. Cell.

[64] Lopez-Acevedo M., Grace L., Teoh D., Whitaker R., Adams D.J., Jia J., Nixon A.B., Secord A.A. (2014). Dasatinib (BMS-35482) potentiates the activity of gemcitabine and docetaxel in uterine leiomyosarcoma cell lines. Gynecol. Oncol. Res. Pract..

