# Supplementary material for: PARP Inhibition Increases the Reliance on ATR/CHK1 Checkpoint Signaling Leading to Synthetic Lethality—An Alternative Treatment Strategy for Epithelial Ovarian Cancer Cells Independent from HR Effectiveness
Source: Int J Mol Sci. 2020 Dec 19;21(24):9715. doi: 10.3390/ijms21249715 (PMC7766831; doi:10.3390/ijms21249715)
Supplement: Supplementary file 1 [file ijms-21-09715-s001.pdf]

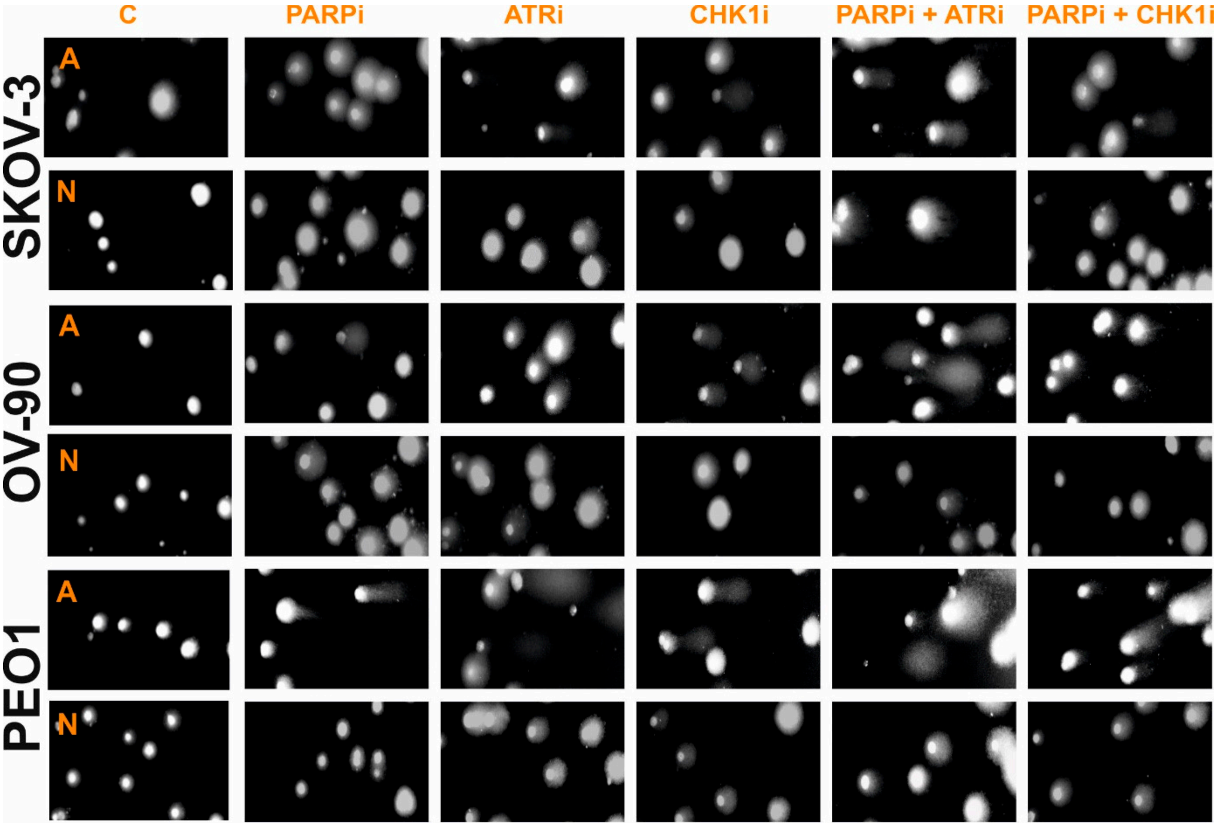

Figure S1. Representative images of the DNA damage in EOC cell lines treated with PARPi, ATRi and CHK1i alone and in combination, measured as a percentage of the DNA in the comet tail. Comets were visualized under a fluorescence microscope after staining with DAPI. The letter A indicates the alkaline version and N the neutral version of the comet assay. Comet assay: exponentially growing cells were treated the same as in Figure 3 for 48 h and processed for comet assay as described under Materials and Methods. Comet image were obtained by analysis system Lucia-Comet v. 4.51.
